# Challenges with study procedure fidelity when conducting household survey: reports from the field

**DOI:** 10.1186/s13104-019-4500-0

**Published:** 2019-08-07

**Authors:** Morenike Oluwatoyin Folayan, Micheal O. Alade, Elizabeth O. Oziegbe

**Affiliations:** Department of Child Dental Health, Obafemi Awolowo University Teaching Hospitals Complex, Ile-Ife, Nigeria

**Keywords:** Study procedure fidelity, Household survey, Field work, Nigeria

## Abstract

**Objectives:**

The aim of the study was to identify reasons for protocol deviations during conduct of large epidemiological surveys despite training of field workers, validating clinicians, and providing field supervisory support. Enquiries focused on breaches of recruitment procedures, privacy, confidentiality, and informed consent. The case study was a household survey conducted in Ile-Ife, Nigeria.

**Results:**

The study reveals that despite training of field workers, providing supervisory support, and conducting validation exercises, protocol deviation still occurred. Measures to improve internal research validity during the conduct of surveys can minimise but not eliminate protocol deviations. Individual and environmental factors increase the risk for protocol deviation. Individual factors include personal bias against adherence to elements of the protocols, and pressure to meet personal recruitment targets to maximise remuneration. These pressures increase the risk of breaching study participants’ recruitment process. Environmental pressures resulted from low research literacy that made it possible for field workers not to consent participants and for participants not to prioritise privacy. The use of electronic data collection enhanced data security. A key recommendation from the study was that improved field supervision will reduce the risk for protocol violation.

**Electronic supplementary material:**

The online version of this article (10.1186/s13104-019-4500-0) contains supplementary material, which is available to authorized users.

## Introduction

Study-protocol fidelity refers to the collection of research data in a uniform manner for all recruited study participants according to the study plan [1]. The term fidelity has often been used in reference to clinical trials, where interventions are required. Intervention fidelity *means that the intervention was conducted as planned* [1] to maintain the study’s internal validity and enhance its external validity. With adherence to these requirements, the reliability of study outcomes is enhanced [2, 3]. Deviation from the study plan, known as study variance [2], threatens the internal validity of the study. Study variance can result from events that affect the study participants, such as selection bias or social threats to them [1].

A few studies have explored reasons for non-compliance with study protocols. The studies have been limited to clinical and non-clinical trials [4], and most have focused on participants’ reasons for non-adherence to study protocols [5]. Adherence to protocols is necessary to ensure quality data collection [5, 6] and accuracy of conclusions [6]. Deviation from study protocol can be minimized by using strategies, such as developing uniform operating procedures and training and monitoring the research team’s activities [1, 4]. This study aimed to identify reasons for protocol deviations during conduct of large epidemiological surveys, despite the institution of measures to prevent these deviations.

## Main text

This was a qualitative study that tried to determine if there were procedure deviations by trained field workers conducting a large household survey, and reasons for deviations if they occurred. The survey’s aim was to determine the maternal psychosocial factors and oral health behaviors that were risk indicators for early childhood caries in preschool children residing in Ife Central Local Government Areas of Osun State, Nigeria.

Data was collected electronically using an interviewer-administered structured questionnaire. The questionnaire elicited information on the socio-demographic profile of mothers and children, the children’s caries risk profile, mothers’ psychosocial status and oral health status of the mother/child dyads. The height and weight of the children were also measured. Internal validity of the study was ascertained in three ways: review of the study instrument for construct validity by two research clinicians, 3 days’ training of the 24 research assistants, and examiner validation of the five clinicians.

Research assistants recruited for the study had prior experience with collecting data in the community. They spent a minimum of 5 h each for 3 days to learn the study objectives and design, the study questionnaire, and study operating procedures. The assistants also pilot-tested the questionnaire to ensure clarity of statements and determine the time spent in data collection. On the 3rd day, the research assistants had lectures on the ethics of research, research integrity, and effective communication during research interviews, and they attended practice session on effective communication. The five dentists, who conducted physical and clinical examinations, were trained by a consultant pediatric dentist then took inter- and intra-examiner reliability tests.

The field work was conducted from 17th December 2018 to 12th January 2019 when students were on school break; 1549 mother–child dyads were recruited. The study coordinators visited the field once a week to meet with the research assistants, observe interviews and data collection, monitor field workers’ adherence to study protocols, and provide on-site logistic support for the data collection. The mean time to fill each questionnaire was about 22 min. Research assistants were paid $0.68 per questionnaire collected, and the dentists were paid $8.20 per day of work. There were no set daily targets for recruitment of study participants for the field workers. Payments were made at the end of the study after the debrief session. Each field worker also received $2.70 airtime to support team communication.

A week after the conclusion of data collection, a focus group discussion was conducted with nine research-team members, including field workers and dentists. In-depth interviews were conducted with the research-team members, which included a dentist, four top-performing and four lowest-performing research assistants. Performance was judged on the average number of questionnaires completed per day. Table 1 shows the profile of the research team members recruited for the focus group discussion and the key informant interviews.

**Table 1:** Description of focus group participants and key informants interviewed

Code	Sex	Designation	Number of past research conducted
Focus group discussants			
P1	Male	Research Assistant	2
P2	Male	Research Assistant	1
P3	Female	Research Assistant	2
P4	Female	Dental Officer	0
P5	Male	Co-Investigator	1
P6	Male	Dental Officer	0
P7	Female	Research Assistant	5
P8	Female	Research Assistant	2
P9	Female	Research Assistant	10
Key informant interviewees			
001_grp1	Male	Research Assistant, high performer	4
002_grp1	Female	Research Assistant, high performer	5
003_grp2	Male	Research Assistant, poor performer	3
004_grp2	Female	Research Assistant, poor performer	5
005grp1	Male	Research Assistant, high performer	1
006grp2	Male	Research Assistant, low performer	5
007_grp1	Female	Research Assistant, high performer	3
009_grp 2	Male	Research Assistant, low performer	4
008_medical officer	Male	Dental officer	0

A semi-structured guide was used to explore research team members’ reflections on their adherence to the study protocol. They were asked to reflect on their adherence to their study protocol—participants’ recruitment process, informed-consenting process, interviewee privacy and data confidentiality. Finally, the research team members were asked to make recommendations on how to improve the adherence of research field staff to research protocols. The interview was audiotaped.

Transcripts of the audio recorded interviews, focus group discussion and hand-written notes (brief field notes, summary notes, debriefing reports) were inputted into ATLAS.ti software for analysis. A deductive theoretical approach was used for coding, using fixed codes created from emerging ideas from the rich textural documents. Responses corresponding to each study objective were summarized and assigned to the descriptive categories. Relevant quotes also were retrieved from the transcripts (see Additional file 1: Data S1).

### Field workers did not completely adhere to study protocol

Some field workers did not completely adhere to study protocols, although the majority stated they adhered to the protocols on privacy, confidentiality, consent procedure, and recruitment guidelines. Some of their responses include;

*“We were able to adhere to the protocol let’s say 99% as a person I cannot speak for the others but as a person I was able to do that on the field.”(007_grp1)*

*“Study protocol on research is a thing that you have been instructed on before you go out to the field and adhering strictly to research protocols is one of the research ethics that is needed to get a good data. As a Research Assistant, I have always been adhering because I know the consequence of any flaws” (004_grp2)*

*“I adhered to the study protocol up to say 85*–*90% of it. The only thing I had challenge with was most times while I am conducting interview, my mind will have skipped the consent part, then when I remember I will quickly give the consent. Apart from that every other thing I am supposed to do I did.” (IDI 001_grp1)*

### Reasons for non-adherence to research protocols on recruitment

The stated reasons for non-adherence included pressure to interview many respondents in order to earn good income daily and lack of familiarity with the study site prior to study commencement, which increased the chance of teams visiting the same study area twice.

*“During the field work as time went on we were actually recruiting more than one study participant per household because it was becoming difficult to identify children 0*–*5* *years old. So in households where there were more than one child 0*–*5* *years per household,* +*we had to do the two children of the mother” (002_grp1)*

*“We did not adhere to our enumeration areas in the field because it was difficult tracking it so sites were visited twice. For instance there were places we got to and we were told they had been examined while some did not talk because they wanted us to examine them again. Some told us honestly that they want to be examined again. I am sure because of cases like that we might have repeated some interviews.” (002_grp1)*

### Reasons for non-adherence to research protocols on privacy

Respondents stated that they tried to ensure the privacy of research participants even when the participants did not see the need for it. Meeting study participants in their homes also prevented holding private interviews.

*“But there were a few challenges as some of the respondents would say they want to be where people are and did not want any form of privacy.” (002_grp 1)*

*“There was no sensitive question in fact there are some questions while asking a respondent that it is the neighbour that will be answering for them. There was no shy moment so privacy was not really taken seriously”. (P3)*

### Reasons for non-adherence to research protocol on confidentiality

The ability to keep data confidential was enhanced by using electronic devices for collecting participant information. None of the participants reported a breach in confidentiality.

### Reasons for non-adherence to research protocols on informed consent

Adherence to the consent process was not perfect. Reasons for non-adherence included forgetting to administer the consent form before commencing interviews, fatigue and pressure to do as many interviews within the shortest time possible. Not all the research field workers felt it important to get participants’ consent, despite their training on the importance of consent.

*“I actually was forgetting the consent part. We may have been working and not getting study participants recruited. When suddenly we get them, we will be so excited and just go ahead to collect the data forgetting to fill the consent form.” (001_grp1)*

*“In all sincerity once in a while, I did no administer the consent forms. I did a research without giving the respondent a consent form to fill. When I have a willing respondent from the discussion, the willingness could imply that the person has given consent but will not get to sign the paper which is the practical part.” (003_grp 2)*

### Respondents’ recommendations to improve research protocol adherence

Suggestions for improving adherence to research protocols included continual field supervision and monitoring to ensure compliance; giving adequate for data collection in order to reduce the workload and prevent protocol deviation to meet set targets; and providing field workers with adequate remuneration to prevent the pressure for meeting personal income targets.

*“I think monitoring is important. Protocol will always be on paper, but there should be a monitoring process such that people will not be able to digress from the essence of the study.” (001_grp1)*

*“It is just training and monitoring and supervision. We should have supervisors that would always come around to ensure that you are doing what you are supposed to do.” (002_grp1)*

*“It is important to make the people in the communities aware of the study so they know what to expect, prepare their minds for the research and this will improve privacy.” (003_grp2)*

*“Debriefing should happen every 2*–*3* *days to discuss challenges in the field. This can then be resolved before the next field visit.” (002_grp1)*

*“Limit the number of study participants per field worker. This means get a lot more research assistant to conduct the work so that each research assistant will do less work” (006_grp2)*

The study highlights that despite adequate training of field workers, providing supervisory support, and conducting validation exercises, protocol deviation still occurs. Measures to improve internal research validity during the conduct of surveys can minimize but not eliminate protocol deviations. The study reported protocol deviations that resulted from individual factors that increase the risk for protocol deviation combined with environmental factors that promote such breach. Individual factors that increase the risk of breaching research protocols include personal bias against adherence to elements of the protocols, and self-imposed pressure to meet personal recruitment targets to maximise remuneration. These factors create pressure on field workers to breach study participants’ recruitment process. Environmental pressures that prompt protocol deviations include poor research literacy, which makes study participants pay little attention to consenting and privacy. The use of electronic data collection methods enhances data security. Training that emphasizes the importance of study-procedure fidelity and measures to ensure it, reduces the risk of protocol deviation.

A strength of this study was the short interval between the time that data collection was completed and the interviews and focus group discussions were conducted, which reduced the risk for recall bias [7]. A lesson highlighted by this study is the need for more stringent oversight support for field workers on the field. Daily supervision and the opportunity to debrief frequently can enable team leads identify unreported protocol deviations early and resolve field problems that may affect data integrity. An example of a field problem in this case study was the potential for double enrolment of study participants, resulting from poor mapping of enumeration sites.

While the training offered to staff may have improved field data collection procedure, the training conducted did not identify the possibility for personal research-related bias to negatively affected study protocol fidelity. This implies that in future, training sessions for field workers should dedicate a session to identifying individual research related bias through value clarification exercises, and address these bias prior to field work. In addition, the daily number of study participants’ recruitment per field worker may need to be fixed at an appropriate number that gives enough time to ensure study protocol adherence. The environmental pressures identified can be mitigated through appropriate staff support (field supervision, debriefs, reiteration of data collection procedures).

## Limitations

The study participants were the judge of their own work, which may have resulted in under-reporting of breaches of study protocol.

## Additional file

**Additional file 1: Data S1.** Transcript of interview and focus group discussions conducted with field workers involved with the household survey.

## Data Availability

Study related data and materials are accessible on request from the lead author.
