# Supplementary material for: Challenges with study procedure fidelity when conducting household survey: reports from the field
Source: BMC Res Notes. 2019 Aug 7;12:493. doi: 10.1186/s13104-019-4500-0 (PMC6686494; doi:10.1186/s13104-019-4500-0)
Supplement: Supplementary file 1 — Additional file 1: Data S1. Transcript of interview and focus group discussions conducted with field workers involved with the household survey. [file 13104_2019_4500_MOESM1_ESM.docx]

**Analysis of Data on Protocol Adherence**

**Methodology**

A total of Nine (9) In Depth Interviews and One (1) Focus Group Discussion comprising Nine (9) Participants were transcribed and inputted into ATLAS.ti software which was used for Analysis.

One Hermeneutic unit was created comprising of Ten (10) Primary documents from the Focus Group Discussion and In Depth Interviews. A deductive theoretical approach was implemented for coding, working with already fixed codes created from emerging ideas from the rich textural documents.

For the analysis, the Network View Manager facilitated the creation of maps composed of codes, memos and quotations.

**Findings**

The study revealed that Field workers do not completely adhere to study protocols in its implementation on the field despite training prior to field work.

On the average, majority of the Respondents said they adhered to the study protocol to a great extent (subjectively rated to be over 85% by many of the respondents) in terms of Privacy, Confidentiality, Consent procedure and Recruitment guidelines.

*“You will agree with me that it’s not about the doctors alone, it is about the doctors and the RAs, but on my own part I tried to do it accordingly because really there is nothing much. I think I followed the protocol, I am not sure I did something that was not in the protocol.” (IDI Dental Officer)*

*“We were able to adhere to the protocol let’s say 99% as a person I cannot speak for the others but as a person I was able to do that on the field.”(IDI 007_grp1)*

*“I wouldn’t judge myself 100%, but I will say to a very large and reasonable extent, I adhered. The consent form, the privacy, the confidentiality, I tried to communicate as much as I can to a large extent. ”IDI 003_grp2”*

*“Study protocol on research is a thing that you have been instructed on before you go out to the field and adhering strictly to research protocols is one of the research ethics that is needed at all levels in order to get a good data. As a Research Assistant, I have always been adhering because I actually have the basic knowledge of this thing and I know the consequence of any flaws” (IDI 004_grp2)*

Reasons for incomplete adherence to research Protocols on Recruitment identified from the data include;

- Insufficient number of study participants to meet the sample size for each study group due to unavailability or lack of interest in participation.
- Lack of or inadequate means to identify areas or persons already visited and interviewed leading to multiple recruitments in a location.
- Pressure to interview a set target number of respondents in order to earn a reasonable amount daily.
- Omission or inadequacy of prior assessment visits to study sites to ensure recent and up to date information about site is gotten in order to plan a successful implementation of investigation that is devoid of many changes to the research on the field that will minimize non adherence to study protocol.

*“During the field work as time went on we were actually doing more than one per household like if a mother has more than one child in the same age grouping. We did more than one 0-5years per household and that is true because when we saw what it was becoming, it was not easy to get 0-5year olds firstly so we had to do that especially when we have twins we had to do the two children of the mother” (IDI 002_grp1)*

*“As per recruiting respondents, it got to a time that when we get to a house whatever children that you see there you just do them, irrespective of the fact that you are not supposed to do two 0-5 per a mother so sometimes you can just do if you see like 40 children because there was a time target”(IDI 006_grp2)*

*“Firstly, as regards the selection of the households I don’t think it was a fair selection because I feel like if there was an enumeration area for each team that this is where you must cover, you must not go outside this enumeration area, people would be able to know that this is the enumeration area they have touched. For instance there were some places we got to they would say they have done for us, some will not talk, some will even want to do it again they will not tell you, some will say honestly I want to do it again and I am sure because of cases like that we might have repeated in this survey areas where people have already done before.” (IDI 002_grp1)*

*“We had some issues, logistics basically and which can affect the study program. There are some locations they gave us that when we get there these places no longer exist, if some of the information is properly updated we will not have any issue.” (IDI 009_grp2)*

About adherence to the study protocol on Privacy, Respondents generally said they tried to ensure the privacy of the research participants to the best of their ability despite circumstances where the participants did not see the need to be interviewed alone. The household settings in which the study was carried out also did not allow for exclusively private interviews.

*“But there were a few challenges some of the respondents would say they want to be where people are they want us to take it there and there were issues that when we were just asking them questions some people would just be talking and we had to just tell them that please let it just be with the person that we are responding with.” (IDI 002_grp 1)*

*“Sometimes their friends can be available but I made the discussion very low like there are some sensitive questions that are very confidential so you gently whisper without making the others around to hear because it wasn't very easy taking them out of that place.” (IDI 009_grp2)*

*“There was no sensitive question in fact there are some questions while asking a respondent that it is the neighbour that will be answering for them. There was no shy moment so privacy was not really taken seriously”. (FGD Participant 3)*

*“Privacy was okay but there were some that the mother will never allow you take the child away so they have to be where they can even hear and see but when we talk about 0-5years we can’t be talking about privacy”. (FGD Participant 9)*

*“I maintained privacy, but there was a woman that said she wants to know what I was asking, I allowed her because there was no emotional question.” (IDI 005_grp1)*

The Respondents were confident they absolutely adhered to keeping information from the study confidential. They were certain the electronic devices used for the study ensured that participant information stayed with only the research team hence protecting participant’s data.

*“For the past experience we were able to keep their secrets likewise this present one we were able to do that also.” (IDI 007_grp1)*

*“This data was collected with tablets on the field and practically, nobody has access to it except the interviewer.” (IDI 003_grp2)*

Adherence to the study protocol with regards to the consent process was still not perfect despite training as respondents gave reasons to include sometimes forgetting to take an informed consent before commencing interviews, fatigue and pressure to do as many interviews within the shortest time possible.

Not all the research field workers see the absolute importance of taking an informed consent despite training.

*“I adhered to the study protocol up to say 85-90% of it, the only thing I had challenge with was most times while I am conducting interview, my mind will have skipped the consent part, then when I remember I will quickly give the consent. Apart from that every other thing I am supposed to do I did.” (IDI 001_grp1)*

*“Why I digressed from the protocol was because those children you don’t normally meet their parents at home to seek consent, so ask for consent from the elderly people around or whom they are staying with because in the study site, there is a specific number of respondents one has to get and most parents are not around.” (IDI 004_grp2)*

*“Maybe because they have a target and they have a lot of people to interview and that there is no much time, because there are some people that in the morning as they start and are very fresh they can talk about the consent but later on in the afternoon may be they are tired they just skip that part but notwithstanding we still talk about it but not in full.” (IDI 006_grp2)*

*“I interviewed one child, the parents were in the house she didn't come out but the child was very fluent. Even though the protocol says we should take the parent’s consent first it doesn’t happen like that all the time.” (IDI 009_grp2)*

*“I actually was forgetting the consent part. Most times while I was working especially when we started doing 0-5years, we may have been working all along and not getting the sample size that we need, and suddenly we get them, we will be excited and just go ahead so it is when I get to the middle that I now discovered I was supposed to have taken this consent.” (IDI 001_grp1)*

*“There was a particular case that I wasn’t there at that moment I was with another RA, the RA told the mother that we are going to ask questions then we are going to look into the child’s mouth, and we are going to look into her own mouth, I think the RA omitted that they will check the child’s mouth, so when I opened the mouth and in fact I had already seen what I needed to see then the mother asked why I checked her child’s mouth so I apologized and said there is even one thing that I need to tell her, she said no, that I should not bother but then I told her that it very good for her and that I was ready to give her a referral letter and she said no, so after then we apologized and left and the RA said we should go ahead and I said no we are not going to go ahead since she didn’t give us full consent. So I ensured the data was deleted.” (IDI Dental Officer)*

*“We used a tablet so we just read out the details of our work so they give a verbal consent and if they refused we move to the next house, we do another random selection ballot to pick the next house.”(IDI 002_grp1)*

*“In all sincerity once in a while, when I am short of consent forms, I did a research without giving the respondent a consent form to fill, when I have a willing respondent from the discussion, it is obvious that the person is willing to participate and it could be implied that the person has given consent but on paper which is the practical part the person could not get to sign it.” (IDI 003_grp 2)*

The Respondents recommended suggestions to ensure full implementation of study protocols by field workers to include;

- More time spent during training to explain the entire research with its objectives and protocol.
- Continuous supervision and monitoring of field work to ensure compliance with research protocol.
- Sufficient time for data collection in order to reduce workload to avoid manipulations in order to meet set targets.
- Good remuneration for field workers to avoid the personal need to increase workload that can compromise research quality through non adherence with the research protocol.

*“I think, more time should be given especially when it has to do with training, especially in driving down the aims and objectives of the research, because it is very important, if we don’t understand what the study is all about, RAs may not really get it, and may not communicate well with the respondents. I think monitoring is important, monitoring is key for that aspect, protocol will always be on paper, but there should be a monitoring process such that people will not be able to digress from the essence of the study.” (IDI 001_grp1)*

*“I think it should be structured in a way to make everybody and the Research Assistants adhere to the protocol. Train the Research Assistants for the purpose of the protocol and the consequences.” (IDI 004_grp2)*

*“Everything still boils down to training, I think without training there is no other way, it is just training and monitoring and supervision. We should have various supervisors like the field work that we did, apart from the RAs we had a supervisor for the RAs for the particular area that would always come around to ensure that you are doing what you are supposed to do.” (IDI 002_grp1)*

*“For the privacy aspect, emphasize it in the training to those people that will carry out the research. What I am saying about the media, is making the people in the communities aware of the study so they know what to expect, preparing their minds that these people are coming and they will be able to allow them some privacy.” (IDI 003_grp2)*

*“There should be a Supervisor for each team not a Doctor but a supervisor that will make sure that things go well that people don’t go out of the protocol. At the end of every survey and if possible it may not even be at the end of every survey may be at the end of every 2-3 days let us all sit and say what are the challenges just about 10/15 minutes. For instance you can report to the supervisor that this is the challenge we are facing and the supervisor can work on it before the next time of field visit.” (IDI 002_grp1)*

*“Consistent monitoring and evaluation and motivation of staff are very important to ensure good protocol adherence. (IDI 009_grp2)*

*“For the RAs I will recommend that on further works they should invest more on Supervisors and not just leave RAs to work alone and assume that they are working fine. There should be Supervisors assigned to each team or group.” (FGD Participant 8)*

*“One thing I think we should do is the number of respondents that a person will be interviewing should be kept small. I think it is better to interview small respondents and be paid small money, may be normally it is going to take like 30 days and the respondents the researcher is going to interview like 300 people instead of recruiting 30 researchers (RAs) they should recruit like 50 or 100 and pay may be 20,000 Naira and if they can do twenty in all that will be better to ensure quality,” (IDI 006_grp2)*

*“There was a time that we really like drifted from the rules because we were given a target for the day but considering the fact that the days were approaching and we have not met up we had to just make some manipulation and stuff like that but it was not really much .This is one thing if the resources provided are sufficient enough the manipulation aspect will be reduced to an extent.” (FGD Participant 3)*

*“I think that it is the target you have to meet and the pay you get that was one big factor that made people digress from the protocol.” (FGD Participant 4)*

*“Basically I won’t say this is because I like money I will say it because remuneration is important, and money makes people eager to work better, the financial aspect of people that will do research must be considered and be factored in well, they must be well paid, they should be given value for the work they do not because it is to be paid, but because it is to be valued.” (IDI 003_grp2)*
